# Noninvasive Visualization of MicroRNA-16 in the Chemoresistance of Gastric Cancer Using a Dual Reporter Gene Imaging System

**DOI:** 10.1371/journal.pone.0061792

**Published:** 2013-04-17

**Authors:** Fu Wang, Xinxing Song, Xiujuan Li, Jing Xin, Shenxu Wang, Weidong Yang, Jing Wang, Kaichun Wu, Xiaoyuan Chen, Jimin Liang, Jie Tian, Feng Cao

**Affiliations:** 1 School of Life Sciences and Technology, Xidian University, Xi'an, Shaanxi, China; 2 Department of Cardiology, Xijing Hospital, Fourth Military Medical University, Xi'an, Shaanxi, China, Xijing Hospital, Fourth Military Medical University, Xi'an, Shaanxi, China; 3 Department of Nuclear Medicine, Xijing Hospital, Fourth Military Medical University, Xi'an, Shaanxi, China; 4 Department of Digestive Disease, Xijing Hospital, Fourth Military Medical University, Xi'an, Shaanxi, China; 5 Laboratory of Molecular Imaging and Nanomedicine, National Institute of Biomedical Imaging and Bioengineering, National Institutes of Health, Bethesda, Maryland, United States of America; 6 Institute of Automation, Chinese Academy of Sciences, Beijing, China; University of Navarra, Spain

## Abstract

MicroRNAs (miRNAs) have been implicated to play a central role in the development of drug resistance in a variety of malignancies. However, many studies were conducted at the *in vitro* level and could not provide the *in vivo* information on the functions of miRNAs in the anticancer drug resistance. Here, we introduced a dual reporter gene imaging system for noninvasively monitoring the kinetic expression of miRNA-16 during chemoresistance in gastric cancer both *in vitro* and *in vivo*. Human sodium iodide symporter (hNIS) and firefly luciferase (Fluc) genes were linked to form hNIS/Fluc double fusion reporter gene and then generate human gastric cancer cell line NF-3xmir16 and its multidrug resistance cell line NF-3xmir16/VCR. Radioiodide uptake and Fluc luminescence signals *in vitro* correlated well with viable cell numbers. The luciferase activities and radioiodide uptake in NF-3xmir16 cells were remarkably repressed by exogenous or endogenous miRNA-16. The NF-3xmir16/VCR cells showed a significant increase of ^131^I uptake and luminescence intensity compared to NF-3xmir16 cells. The radioactivity from *in vivo* ^99m^Tc-pertechnetate imaging and the intensity from bioluminescence imaging were also increased in NF-3xmir16/VCR compared with that in NF-3xmir16 tumor xenografts. Furthermore, using this reporter gene system, we found that etoposide (VP-16) and 5-fluorouracil (5-FU) activated miRNA-16 expression *in vitro* and *in vivo*, and the upregulation of miRNA-16 is p38MAPK dependent but NF-κB independent. This dual imaging reporter gene may be served as a novel tool for *in vivo* imaging of microRNAs in the chemoresistance of cancers, as well as for early detection and diagnosis in clinic.

## Introduction

Gastric cancer remains the first leading cause of cancer death in China and the fourth most common malignancy worldwide despite a dramatic decrease in its mortality and morbidity over the past three decades [1]. Chemotherapy has been widely used to treat both resectable and advanced gastric cancer, leading to improvements in overall survival as well as quality of life for patients [2], [3]. However, long-term chemotherapy often fails to eliminate all tumor cells because of intrinsic or acquired multidrug resistance (MDR), which is the most primary cause of tumor recurrence [4]. At present, the MDR has been considered as a multifactorial phenomenon involving several main mechanisms, including increased metabolism of drugs, decreased uptake of water-soluble drugs, altered drug targets, reduced intracellular drug concentration by efflux pumps, altered cell cycle checkpoints and induced emergency response genes to impair apoptotic pathways, *etc* [5]. Although the MDR mechanisms have been extensively explored, the key determinants of this phenomenon remain largely unclear.

MicroRNAs (miRNAs) are a novel class of endogenous small noncoding RNAs (19–23 nucleotides) that negatively regulate gene expressions at the post-transcriptional level by perfect or imperfect base pairing with the 3′ untranslated region (UTR) of target mRNAs and thereby induce mRNAs degradation or translation repression [6]. Currently, emerging studies have suggested the existence and importance of miRNAs in the evolution of anticancer drug resistance and miRNAs expression profiling can be correlated with the development of drug resistance [7]–[10], indicating that the miRNAs-mediated form of drug resistance adds another mechanism of MDR. In our previous study, it was reported that two miRNAs, miRNA-15b and miRNA-16, were differentially expressed in a multidrug-resistant human gastric cancer cell line SGC7901/VCR and its parental cell line SGC7901 [11]. However, it was conducted only at the *in vitro* level and could not reflect the *in vivo* information on the functions of miRNAs in the anticancer drug resistance.

Recent advances in molecular imaging techniques allows the noninvasively visualization of normal and abnormal cellular processes in living subjects at the molecular level rather than at the anatomic level [12]. Several noninvasive strategies, such as using a fluorescent protein, luciferase reporter gene, nucleolin aptamer or fluorescent molecular beacon conjugated nanoparticle, have been developed to monitor the expression patterns of various miRNAs during carcinogenesis, neurogenesis or myogenesis *in vitro* and *in vivo* [13]–[16]. The present study was to introduce a noninvasive method for monitoring miRNA-16 in the chemoresistance of gastric cancer through a dual imaging reporter gene system in which human sodium iodide symporter (hNIS) and firefly luciferase (Fluc) genes were linked to a fusion gene for bioluminescence imaging and ^99m^Tc-pertechnetate gamma camera imaging *in vivo*.

## Materials and Methods

### Animals

Specific pathogen-free 8-week-old female BALB/c nude mice were obtained from the Shanghai Animal Center in China and bred in the Fourth Military Medical University animal center, China. All experimental animals were housed under specific pathogen-free conditions. The animal protocols used in this study were approved by the Fourth Military Medical University Ethics Review Board. All procedures were performed in accordance with the Fourth Military Medical University Guide for the Care and Use of Laboratory Animals formulated by the National Society for Medical Research.

### Reagents and antibodies

Mouse monoclonal antibody against hNIS (ab17795) was purchased from Abcam. Rabbit polyclonal antibody specific to Bcl-2 was purchased from Santa Cruz Biotechnology (Santa Cruz, CA). Bay11-7082 (NF-κB inhibitor) and SB203580 (p38 MAPK inhibitor) were obtained from Beyotime Institute of Biotechnology (Shanghai, China). The anticancer drugs including vincristine (VCR), etoposide (VP-16), mitomycin C (MMC), cisplatin (CDDP), 5-fluorouracil (5-FU) and Adriamycin (ADR) were purchased from Wolsen Biotechnology (Xi'an, China). The GV260-Fluc-puro lentivirus plasmid was obtained from Shanghai GeneChem Company (Shanghai, China). All cell culture media and serum were purchased from Gibco (Grand Island, NY).

### Dual reporter gene plasmid construct

The coding sequence of hNIS gene was amplified by PCR from a plasmid kindly provided by Dr. Weidong Yang (Fourth Military Medical University, China) using the following primers:

hNIS-Fw: 5′-CGGGATCCCGCCACCATGGAGGCCGTGGAGACC-3′

hNIS-Rev: 5′-CGGGTACCGGTACGAGGTTTGTCTCCTGCTGGTC-3′

**Figure 1:**
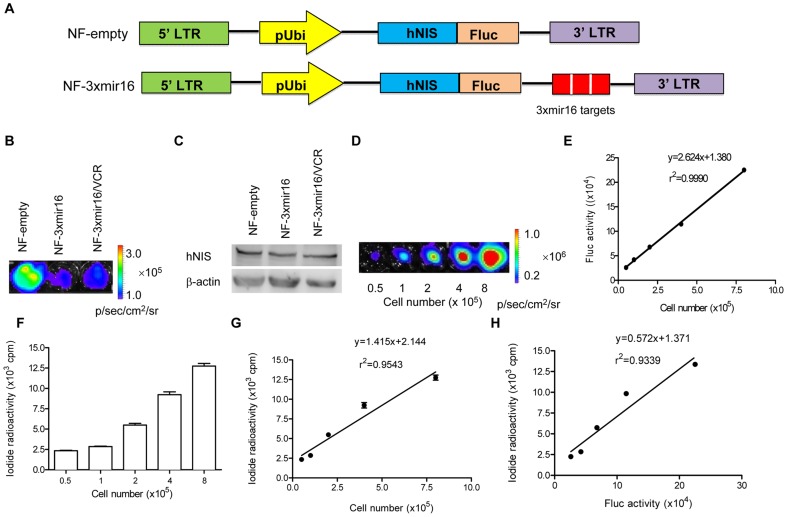
*In vitro* analysis of hNIS/Fluc expression in gastric cancer cell lines. (A) Scheme of reporter gene system in which hNIS and Fluc gene were fused generate NF-empty construct (top). Three copies of complementary sequences against miRNA-16 (3xmir16 targets) were inserted after the hNIS/Fluc fusion gene to obtain NF-3xmir16 construct (bottom). (B) Equal numbers of three types of cells (1×10^5^) were seeded and followed by *in vitro* bioluminescence imaging to detect Fluc expression. Results shown are representative of 3 independent experiments. (C) Western blotting to detect hNIS expression with anti-hNIS (1∶ 1000) or anti-β-actin (1∶ 2000) antibodies. β-actin was used as internal control. Results shown are representative of 3 independent experiments. (D) Luminescence intensity according to cell number. (E) Linear regression analysis between luminescence intensity and cell number. (F) ^131^I uptake assay according to cell number. (G) Linear regression analysis between ^131^I uptake and cell number. (H) Linear regression analysis between bioluminescence intensity and radioactivity.

For the construction of a dual expression vector, the cDNA of hNIS gene was inserted into the *BamH* I and *Age* I restriction enzyme sites of a lentiviral vector GV260-Fluc-puro (Shanghai GeneChem, China) that encodes a Fluc gene under the control of the ubiquitin promoter. The resulting dual expression construct GV260-hNIS/Fluc was further used to generate GV260-hNIS/Fluc-3xmir16 plasmid. Three copies of complementary sequences against miRNA-16 (3xmir16) were inserted after the stop codon of the hNIS/Fluc fusion gene to generate GV260-hNIS/Fluc-3xmir16 (Figure 1A). A scrambled nucleotide sequence of similar length to 3xmir16 was also inserted at the 3′UTR of hNIS/Fluc fusion gene to obtain a control construct (GV260-hNIS/Fluc-scramble. The 3xmir16 sequence or scramble sequence were obtained by annealing the following oligonucleotides:

3xmir16-Fw: 5′-CGCCAATATTTACGTGCTGCTACGCCAATATTTACGTGCTG-CTACGCCAATATTTACGTGCTGCTA-3′

3xmir16-Rev: 5′-TAGCAGCACGTAAATATTGGCGTAGCAGCACGTAAATAT-TGGCGTAGCAGCACGTAAATATTGGCG-3′

Scramble-Fw: 5′- TCGTGGCGTCATTTCTTCAGAATCCGTCCTCCCAAGTACT-CAGTGTGCTAATGTCTATCGATACAA-3′

Scramble-Rev: 5′-TTGTATCGATAGACATTAGCACACTGAGTACTTGGGAGG-ACGGATTCTGAAGAAATGACGCCACGA

### Gastric cancer cell culture and stable cell line generation

Human gastric cancer cell line SGC7901 and its multidrug-resistant variant SGC7901/VCR (established and maintained in our laboratory as previously described [11]) were cultured in RPMI-1640 medium supplemented with 10% fetal calf serum and 100 units penicillin and 100 µg/mL streptomycin (Invitrogen). To maintain the MDR phenotype, vincristine (with final concentration of 1 µg/ml) was added to the culture medium for SGC7901/VCR cells. To generate stable cell line, lentivirus vector GV260-hNIS/Fluc or GV260-hNIS/Fluc-3xmir16 was first cotransfected into 293T cells with packaging plasmids (*gag, pol, vsv-g*). Growth medium was changed at 6 h post-transfection and lentivirus-containing supernatant was harvested at 48 h post-transfection. Harvested supernatants were centrifuged at 4,000 g for 10 min to pellet cell debris. Concentrated virus was titrated on 293T cells. SGC7901 cells and SGC7901/VCR cells were transduced with GV260-hNIS/Fluc and GV260-hNIS/Fluc-3xmir16 virus respectively at a multiplicity of infection (MOI) of 10. Then the cells were screened with puromycin for 3 weeks and cell colonies were collected to expanded for next step experiments.

### RNA oligos and transfection

The miRNA-16 and negative control (NC) RNA oligos were synthesized (Shanghai GenePharma, China) by using the following sequences:

miRNA-16 sense: 5′-UAGCAGCACGUAAAUAUUGGCG-3′

miRNA-16 anti-sense: 5′-CGCCAAUAUUUACGU-GCUGCUA-3′

NC sense: 5′-UUGUACUACACAAAAGUACUG-3′

NC anti-sense: 5′-CAGUACUUUUGUGUAGUACAA-3′

Before transfection, SGCC7901 cells were seeded at 1×10^5^ cells per well in a 24-well plate and grow for 24 h. Transfection was performed with Lipofectamine 2000 reagent (Invitrogen) according to the manufacturer's protocol. All transfection were carried out in triplicate.

### Quantitative real time PCR (qRT-PCR) analysis

Total RNA was isolated from the cultured cells using Trizol (Invitrogen). RT was performed with PrimerScript RT Regent Kit (TaKaRa, Japan) and qPCR was performed with Fast SYBR Green Master Mix (Applied Biosystems, Foster City, CA) and ABI StepOne Plus Real-time PCR system (Applied Biosystems) according to the manufacturer's protocol. PCR products were analyzed on 3% agarose gels. The relative amount of miRNA-16 was normalized to U6 snRNA. And the relative amount of Fluc was normalized to GAPDH gene. The fold-change for miRNA-16 or Fluc gene from experiment group relative to the control group was calculated using the 2^−ΔΔCt^ method, where ΔΔCt = ΔCt experiment - ΔCt control and ΔCt = Ct miRNA - Ct U6 or ΔCt = Ct Fluc - Ct GAPDH. PCR was performed in triplicate. The primers used for stem-loop RT-PCR for miR-16 are listed as follows:

U6 Fw: 5′-GCTTCGGCAGCACATATACTAAAAT-3′

U6 Rev: 5′-CGCTTCACGAATTTGCGTGTCAT-3′

miR-16 RT 5′-GTCGTATCCAGTGCGTGTCGTGGAGTCGGCAATTGCACTGG-ATACGACCGCCAAT-3′

miR-16 Fw: 5′-TAGCAGCACGTAAATATTGGCG-3′

miR-16 Rev: 5′-TGCGTGTCGTGGAGTC-3′

FLuc–Fw: 5′-GGTCCTATGATTATGTCCGGTTATG-3′

FLuc-Rev: 5′-ATGTAGCCATCCATCCTTGTCAAT-3′

GAPDH-Fw: 5′-GGTCTCCTCTGACTTCAACA-3′

GAPDH-Rev: 5′-AGCCAAATTCGTTGTCATAC-3′

### Western blot analysis

SGC7901 cells were washed three times with PBS and then lysed in cold lysis buffer (20 mM NaCl, 200 mM Tris-HCl [pH 7.4], 1 mM phenylmethylsulfonyl fluoride, 1% Triton X-100 and 1% aprotinin) for 30 min on ice. Cell lysates were then centrifuged at 12,000 rpm for 15 min at 4°C. The supernatant was collected and identical amounts of protein were resolved by 8% SDS-PAGE and then transferred to nitrocellulose membranes. The membranes were incubated in blocking solution containing 5% BSA for 1 h at room temperature, and then immunoblotted with indicated antibodies. Immunoreactive bands were visualized using an enhanced chemiluminescence kit (Amersham Pharmacia Corp, Piscataway, NJ).

### MTT assay

To determine the growth rates of NF-empty, NF-3xmir16 and NF-3xmir16/VCR cells, these three types of cells were inoculated into 96-well plates with the same numbers (5,000 cells) per well. At different time points (24, 48, 72, 96 h), 25 µl of 5.0 mg/ml sterile filtered 3-(4, 5-dimethylthiazol-2-yl)-2, 5- diphenyl tetrazolium bromide (MTT; Sigma) was added to each well. The unreacted dye was removed after 4 h incubation, and the insoluble formazan crystals were dissolved in 100 µl of dimethylsulfoxide (DMSO). The absorbance at 570 nm (reference wavelength, 630 nm) was measured with a Synergy II multimode microplate reader (BioTech Instruments, VT).

### Radioactive iodide uptake assay

To identify the relationship between iodide uptake and cell number, a dilution series of cells were inoculated into 24-well plates. After 12 h incubation, the ^131^I uptake level was examined. The iodide uptake was determined by incubating the cells with 500 µL Hanks' balanced salt solution (HBSS) containing 0.5% bovine serum albumin with 37 kBq of carrier-free Na^131^I and 10 mM NaI for 30 min. After incubation, the cells were washed twice as quickly as possible with 1 mL of ice-cold HBSS buffer. Cells were detached with 200 µL trypsin, and the radioactivity was measured using a γ-counter (Perkin-Elmer). To evaluate the functional expression of hNIS in reporter gene system, NF-3xmir16 cells were seeded in at 1×10^5^ cells per well in a 24-well plate the day before transfection, then miRNA-16 and NC oligos were transfected. 24 h later, the iodide uptake were measured as described above.

### *In vitro* bioluminescence imaging assay

To identify the relationship between luminescence signals and cell numbers, a dilution series of cells were inoculated into 24-well plates. After 12 h incubation, each well was washed with phosphate-beffered saline (PBS). Then D-Luciferin (Xenogen) at a concentration of 0.5 mmol/L was added immediately before assay. Bioluminescence was measured with an IVIS 100 Imaging system (Xenogen) and analyzed using the Living Image software version 2.50 (Xenogen). To evaluate the functional expression of Fluc in reporter gene system, NF-3xmir16 cells were seeded in at 1×10^5^ cells per well in a 24-well plate the day before transfection, then miRNA-16 and NC oligos were transfected. 24 h later, the bioluminescence assay was measured as described above.

### Bioluminescent and ^99m^Tc-pertechnetate imaging in nude mice

Equal numbers (5×10^6^ cells) of NF-empty and NF-3xmir16, or NF-3xmir16/VCR and NF-3xmir16 cells, were xenografted subcutaneously into the left and right hind flank of each nude mouse as indicated in results. Bioluminescent imaging was acquired one day after cell injection. All mice were anesthetized with 2.5% isoflurane gas in oxygen at a flow of 1.5 L/min. An aqueous solution of D-luciferine (150 mg/kg body weight) was injected percutaneously 10 min before imaging. The whole-body images for Fluc signals were acquired for 2 min and Living Image software (Xenogen) was running to obtain bioluminescent images. A ROI was selected manually over the signal intensity. Bioluminescence signals are expressed as photons per second per cubic centimeter per steradian (p/sec/cm^2^/sr) within an ROI.

For nuclear imaging, the tumor bearing mice were intraperitoneally injected with 18.5 MBq of ^99m^Tc-pertechnetate at 2weeks after cell injection. 30 min after injection, the animal was placed in a spread-supine position on the bed of the SPECT scanner (Symbia T2, Siemens). Anesthesia was performed with 1%–2% isoflurane in 100% O_2_ during injection and imaging. All images were reconstructed with a 2-dimensional ordered-subsets expectation maximum algorithm. Activity was quantified by viewing the regions of interest (ROI) in the tumors and the area of the ROI was kept constant for all nuclear and optical images. After bioluminescent and ^99m^Tc-pertechnetate imaging, the tumors were collected and weighed.

### Statistical analysis

Results are expressed as mean ±SD. Data showing comparisons between two groups were assessed using the Student's t-test. Comparisons between more than two groups were assessed using analysis of variance (ANOVA) with the appropriate posthoc testing. P values <0.05 were considered statistically significant.

## Results

### Establishment of gastric cancer cell lines stably expressing hNIS and Fluc genes

To generate a fusion reporter gene (GV260-hNIS/Fluc, referred to NF-empty), the hNIS cDNA was cloned into a lentivirus vector (GV260-Fluc-puro) encoding a Fluc gene to generate a fusion protein, which was under the control of ubiquitin promoter. Then three copies of complementary sequences against miRNA-16 (3xmir16) were inserted after the stop codon of the hNIS/Fluc fusion gene to generate another construct (GV260-hNIS/Fluc-3xmir16, referred to NF-3xmir16) (Figure 1A). A scrambled nucleotide sequence of similar length to 3xmir16 was also inserted at the 3′UTR of hNIS/Fluc fusion gene to obtain a control construct (GV260-hNIS/Fluc-scramble, referred to NF-scramble). *In vitro* bioluminescence imaging showed that Fluc gene activity from NF-empty cells was similar to that from NF-scramble cells (Figure S1A). So we choose the NF-empty construct in further study. Two cell lines, a MDR human gastric cancer cell line SGC7901/VCR and its parental cell line SGC7901, were transduced with the lentivirus containing the NF-empty or NF-3xmir16 gene respectively to establish three stable cell lines NF-empty/SGC7901, NF-3xmir16/SGC7901 and NF-3xmir16/SGC7901/VCR (referred to NF-empty, NF-3xmir16 and NF-3xmir16/VCR). First we performed MTT assay to measure the growth rates of these three cell lines (Figure S1B). Then we perfomed qPCR assay to investigate the integrated copies of reporter constructs in the three cell lines (Figure S1C). *In vitro* bioluminescence imaging (Figure 1B) and western blotting (Figure 1C) were further performed to demonstrate the successful expression of Fluc and hNIS genes in these three cell lines.

### Linearity correlation of Fluc and hNIS transgene activities with cell numbers *in vitro*

To evaluate the Fluc and hNIS transgene activities in cells, we performed *in vitro* bioluminescence imaging and ^131^I radioiodide uptake assays in a dilution series of the NF-3xmir16 cell lines. As shown in Figure 1D, according to increases in the numbers of cells, the accumulation of luminescence also increased. Bioluminescence signals were found to correlate well with cell numbers (γ^2^ = 0.9990) (Figure 1E). Meanwhile, ^131^I uptake was also increased with cell numbers (Figure 1F). The radioiodide uptake was observed well correlated (γ^2^ = 0.9543) with cell numbers (Figure 1G). Because the Fluc was fused with hNIS, a well correlation between bioluminescence intensity and ^131^I radioactivity was noted in cells according to the linear regression analysis (γ^2^ = 0.9339) (Figure 1H).

### Validation of miRNA-16 activities *via* the reporter gene system

**Figure 2:**
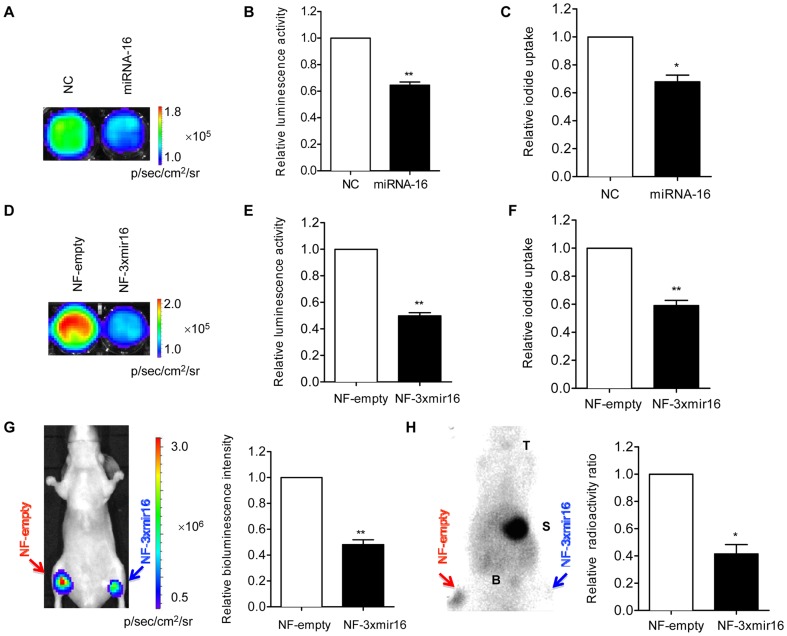
Monitoring of exogenous and endogenous miRNA-16 expression *in vitro* and *in vivo*. (A, B) *In vitro* bioluminescence imaging and (C) radioiodide uptake assay after transfecting miRNA-16 or negative control (NC) RNA oligos (50 nM) into NF-3xmir16 cells. Imaging analysis program (Living Image software version 2.50) was used to quantify the bioluminescence intensity. Triplicate independent experiments were performed for each assay. Data are shown as fold changes in miRNA-16 transfected cells relative to NC transfected cells, which is set as 1. (D, E) *In vitro* bioluminescence imaging of NF-empty and NF-3xmir16 cells with equal numbers (1×10^6^). (F) The relative of radioiodide uptake between NF-empty and NF-3xmir16 cells. Data are shown as fold changes in NF-3xmir16 relative to NF-empty cells, which are set as 1. (G) Equal numbers (1×10^7^) of NF-empty and NF-3xmir16 cells were respectively xenografted into left and right hind limb of each mouse (n = 6). Injection of D-luciferin (150 mg/kg) and then *in vivo* bioluminescence imaging was performed. (H) Injection of ^99m^Tc-pertechnetate (18.5 MBq) and gamma camera imaging was performed. Data are shown as fold changes in NF-3xmir16 xenografts relative to NF-empty xenografts, which are set as 1. ^*^P<0.05, ^**^P<0.005. T = thyroid; S = stomach; B = bladder.

In order to test whether NF-3xmir16 reporter vector containing miRNA-16 target sequences was repressed by exogenous miRNA-16, we examined the Fluc and hNIS activity after introducing miRNA-16. Compared to the cells transfected with NC oligos, the bioluminescence signal were decreased by 35% in the cells transfected with miRNA-16 (Figure 2A and 2B). And the ^131^I radioiodide uptake in miRNA-16 transfected cells was approximately 70% of that in NC transfected cells quantified by radioactive counting (Figure 2C). And the Fluc and hNIS activity were decreased in a dose dependent manner (Figure S1D–F). These data indicated that the NF-3xmir16 reporter construct could be modulated by miRNA-16.

### Detection of endogenous miRNA-16 expression *in vitro* and *in vivo*

To detect the endogenous miRNA-16 expression level in gastric cancer cells *in vitro*, equal numbers (1×10^6^) of NF-empty and NF-3xmir16 cells were seeded and then the Fluc and hNIS activity were examined. As shown in Figure 2D and 2E, Fluc activity resulting from NF-3xmir16 cells was decreased by about 50% by endogenous miRNA-16 compared with that from NF-empty cells. And the hNIS activity from NF-3xmir16 cells was also reduced by about 40% in response to endogenous miRNA-16 in contrast to that from NF-empty cells (Figure 2F). The lack of repression of Fluc or hNIS activities observed in NF-empty cells is in respect that there were no miRNA-16 target sites in NF-empty construct.

The dual imaging reporter gene system enabled *in vivo* visualization of endogenous miRNA-16 expression in gastric cancer cells. Equal numbers (1×10^7^) of NF-empty and NF-3xmir16 cells were xenografted subcutaneously into the left and right hind flanks of each nude mouse (n = 6) and then bioluminescent imaging and ^99m^Tc-pertechnetate gamma camera imaging were acquired. As shown in Figure 2G, luminescence intensity from implanted NF-3xmir16 cells were about 55% lower than that from NF-empty cells, similarly for the difference of luciferase activities measured *in vitro* (Figure 2D and 2E). After the intraperitoneal injection of ^99m^Tc-pertechnetate at 18.5 MBq, gamma camera imaging was performed. In contrast to NF-empty tumors, which showed prominent uptake of ^99m^Tc-pertechnetate, NF-3xmir16 tumors showed negligible uptake (Figure 2H), indicating endogenous miRNA-16 reduced the hNIS expression. Physiologic uptake was also observed at the sites of the thyroid, stomach and bladder, in which hNIS is normally expressed. Regions of interest (ROI) analysis showed that hNIS activity decreased significantly in NF-3xmir16 xenografts compared with that in NF-empty xenografts (Figure 2H). After imaging, we collected and weighed the NF-empty and NF-3xmir16 tumors (Figure S1G). The results showed they had the same weights, indicated that the differences in signal intensity are indeed due to endogenous miRNA-16 function but not cell numbers.

### Visualization of expression change of miRNA-16 in MDR gastric cancer cells *in vitro* and *in vivo*

**Figure 3:**
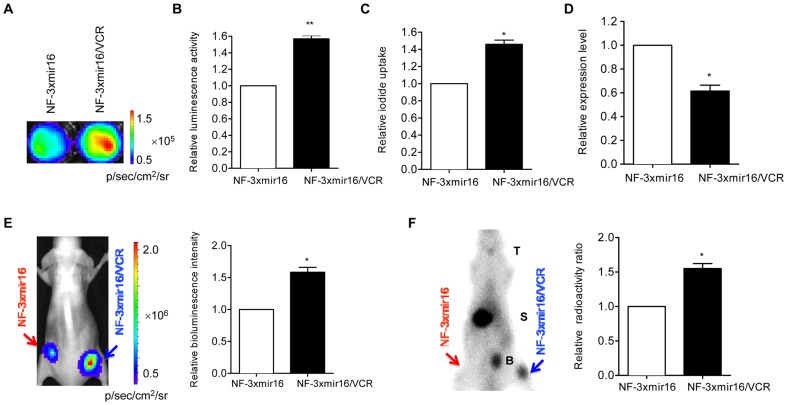
Visualization of differential expression of miRNA-16 in MDR gastric cancer cells. (A, B) *In vitro* bioluminescence imaging of NF-3xmir16 and NF-3xmir16/VCR cells with equal numbers (1×10^6^). (C) The relative of radioiodide uptake between NF-3xmir16 and NF-3xmir16/VCR cells. Data are shown as fold changes in NF-3xmir16/VCR relative to NF-3xmir16 cells, which are set as 1. (D) Quantitative RT-PCR detected miRNA-16 expression in NF-3xmir16 and NF-3xmir16/VCR cells. Triplicate assays were performed for each RNA sample and the relative expression of miRNA-16 was normalized to U6 snRNA. Data are shown as fold change of miRNA levels in NF-3xmir16/VCR relative to NF-3xmir16 cells, which are set as 1. (E) Equal numbers (1×10^7^) of NF-3xmir16 and NF-3xmir16/VCR cells were respectively xenografted into left and right hind limb of each mouse (n = 6). Injection of D-luciferin (150 mg/kg) and then the *in vivo* bioluminescence imaging was performed. (F) Injection of ^99m^Tc-pertechnetate (18.5 MBq) and acquisition of ^99m^Tc-pertechnetate gamma camera imaging. Data are shown as fold changes in NF-3xmir16/VCR xenografts relative to NF-3xmir16 xenografts, which are set as 1. ^*^P<0.05, ^**^P<0.005. T = thyroid; S = stomach; B = bladder.

The expression change of miRNA-16 in drug resistant gastric cancer cells was detected *via* the Fluc and hNIS activity by bioluminescence imaging and ^131^I radioiodide uptake assays. Both Fluc activity (Figure 3A and 3B) and hNIS activity (Figure 3C) increased by about 1.5 fold in NF-3xmir16/VCR cells as compared with that in NF-3xmir16 cells, which suggested that miRNA-16 was downregulated in NF-3xmir16/VCR cells. To verify the results obtained by reporter gene system, quantitative RT-PCR (Q-PCR) was carried out to analysis the differential expression of miRNA-16 in these two cell lines. In accordance with the reporter gene system data, Q-PCR showed decreased miRNA-16 levels in NF-3xmir16/VCR compared to its counterpart NF-3xmir16 cells (Figure 3D).

For noninvasive quantitative monitoring of miRNA-16 differential expression in MDR gastric cancer cells in *vivo*, bioluminescent imaging and ^99m^Tc-pertechnetate gamma camera imaging were performed. The bioluminescent imaging demonstrated that luminescence signals were about 1.7 fold increases in the NF-3xmir16/VCR xenografts versus NF-3xmir16 xenografts (Figure 3E), in accordance with the increases of luciferase activity measured *in vitro* (Figure 3A and 3B). The injection of ^99m^Tc-pertechnetate and the acquisition of gamma camera imaging indicated that a significantly higher accumulation of ^99m^Tc-pertechnetate was observed in NF-3xmir16/VCR xenografts than that in NF-3xmir16 xenografts. Physiological ^99m^Tc-pertechnetate accumulations were also observed in the thyroid gland, bladder and stomach (Figure 3F). And the NF-3xmir16 and NF-3xmir16/VCR tumors had the same weights (Figure S1H).

### VP-16 and 5-FU upregulation of miRNA-16 expression noninvasively imaged by the dual reporter gene system

**Figure 4:**
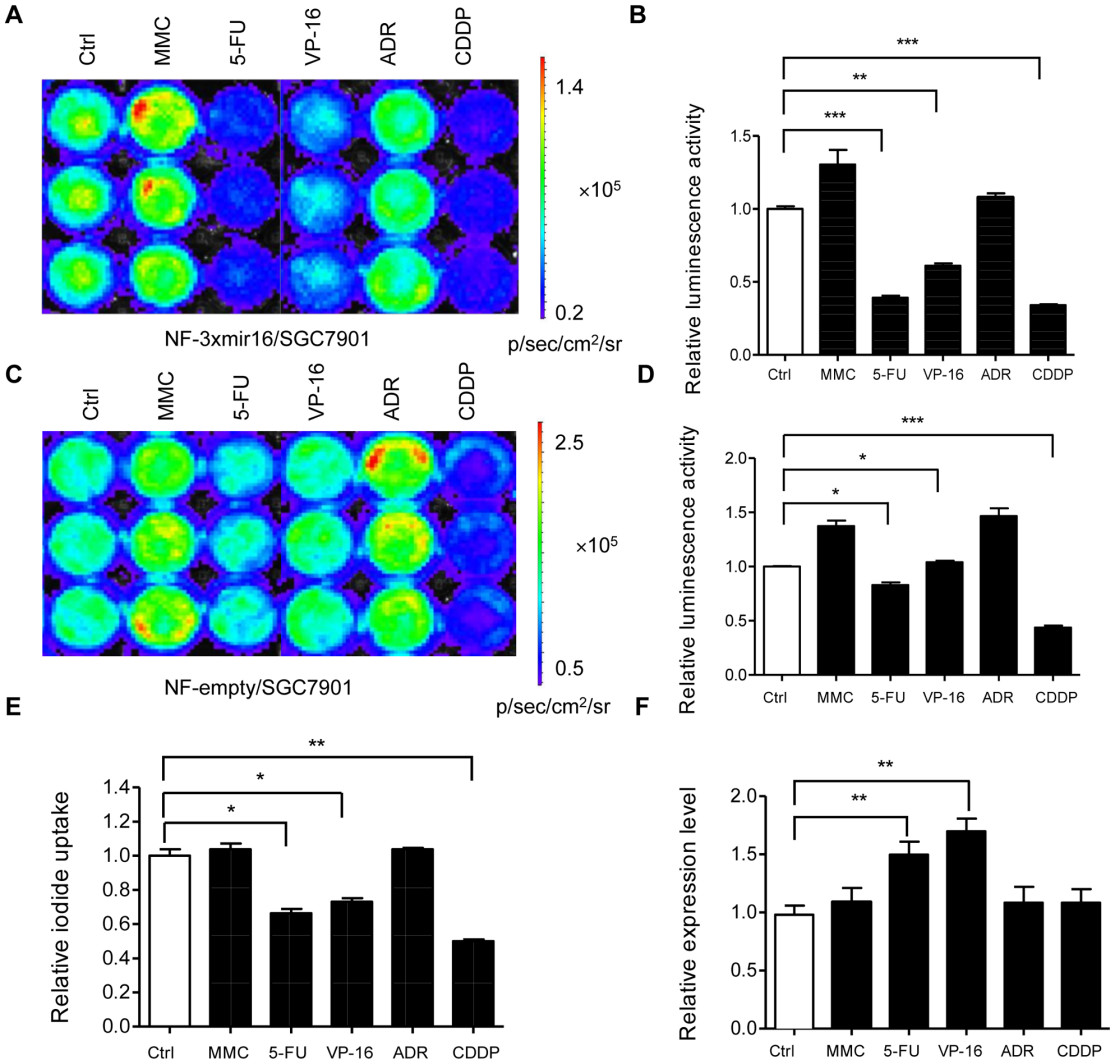
MiRNA-16 was upregulated by VP-16 and 5-FU anticancer drugs. (A, B) NF-3xmir16/cells were treated with VP-16 (5 µg/ml), MMC (0.5 µg/ml), ADR (0.2 µg/ml), 5-FU (10 µmol/L) and CDDP (5 µmol/L), respectively for 48 h and *in vitro* bioluminescence imaging was performed. Results are expressed as mean ± SD of 3 independent experiments. (C, D) NF-empty cells were treated with the above drugs and *in vitro* bioluminescence imaging was performed as described in (A). Results are expressed as mean ± SD of 3 independent experiments. (E) NF-3xmir16 cells were treated with the above drugs for 48 h and radioiodide uptake assay were performed. (F) Quantitative RT-PCR detected miRNA-16 expression in drug treated and untreated NF-3xmir16 cells. Triplicate assays were performed for each RNA sample and the relative expression of miRNA-16 was normalized to U6 snRNA. Results are expressed as mean ± SD of 3 independent experiments. ^*^P<0.05, ^**^P<0.005, ^***^P<0.001 compared with untreated cells.

To determine whether other anticancer drugs could alter miRNA-16 expression profile, five clinical drugs for gastric cancer were added to NF-3xmir16 cells followed by *in vitro* bioluminescence imaging and ^131^I radioiodide uptake assays. The results revealed that the luminescence intensity (Figure 4A and 4B) or the cellular iodide uptake (Figure 4E) were greatly reduced exposure to VP-16, 5-FU and CDDP, but not to MMC and ADR treatment compared to those nontreated cells.

The reasons for the decreased signal observed in VP-16, 5-FU and CDDP treatment might be that the drugs activated endogenous miRNA-16 expression or inhibited cellular growth even caused cell death. To exclude the latter possibility, the five drugs were added to NF-empty cells, which contain no miRNA-16 target sites in the construct. The *in vitro* bioluminescence imaging results demonstrated that VP-16 and 5-FU had no inhibitory effect on the luminescence intensity, whereas CDDP still reduced the luminescence signal (Figure 4C and 4D), indicating that CDDP may inhibit cellular growth but not activate endogenous miRNA-16 expression. On the other hand, MMC and ADR were found to slightly increase the luminescence intensity in both NF-3xmir16 and NF-empty cells (Figure 4A–D), which may result from promoting cellular proliferation.

To verify that miRNA-16 activation participated into anticancer effects from VP-16 and 5-FU, Q-PCR were performed to determine the miRNA-16 level in NF-3xmir16 cells exposure to anticancer drugs treatments. Accordingly, miRNA-16 expression was upregulated significantly after VP-16 or 5-FU treatments in SGC7901 cells compared to the untreated control cells, while MMC, ADR and CDDP had minor effect on miRNA-16 expression (Figure 4F).

### Increased miRNA-16 expressions by VP-16 and 5-FU are involved in p38 MAPK but NF-κB signaling pathway

Similar to the modulation of protein-coding RNA genes, transcription of miRNA genes appears to be regulated by multiple signaling pathways. Activation of the NF-κB or MAPK signaling pathways is a common response following chemotherapeutic drugs [17], [18]. Therefore, we hypothesized that upregulation of miRNA-16 might be a result of increased NF-κB or MAPK activation after VP-16 or 5-FU treatment. To elucidate the role of these signaling pathways in VP-16 and 5-FU-induced transactivation of miRNA-16, we first measured the luminescence intensity in NF-3xmir16 cells in response to VP-16 and 5-FU in the presence or absence of pharmacological inhibitors to NF-κB or p38 MAPK. Treatment of cells with a specific NF-κB inhibitor, Bay 11-7082, had no effect on the luminescence signal compared to the drug treated cells without inhibitors (Figure S2).

**Figure 5:**
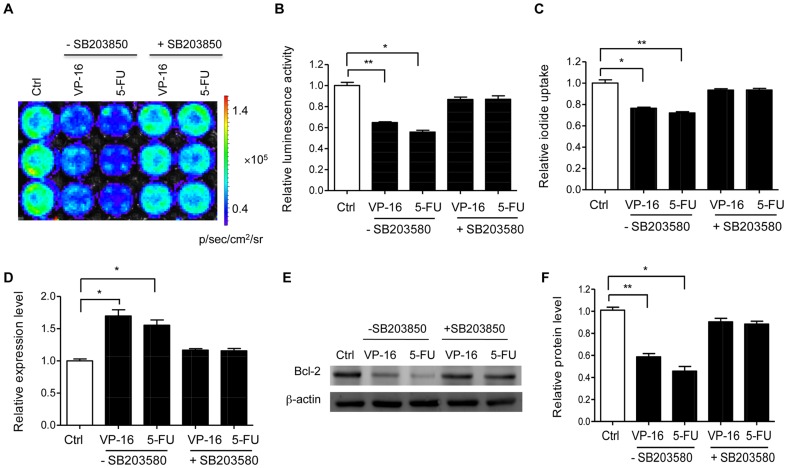
Influence of p38 MAPK signaling pathway on the upregulation of miRNA-16 by VP-16 and 5-FU. NF-3xmir16 cells were pretreated with or without SB203850 (10 µmol/L) for 1 h and then incubated with VP-16 (5 µg/ml) or 5-FU (10 µmol/L) for 48 h. Then *in vitro* bioluminescence imaging (A, B) and radioiodide uptake assay (C) were performed. Results are expressed as mean ± SD of 3 independent experiments. (D) Quantitative RT-PCR detected miRNA-16 expression in NF-3xmir16 cells with or without SB202850. Triplicate assays were performed for each RNA sample and the relative expression of miRNA-16 was normalized to U6 snRNA. Results are expressed as mean ± SD of 3 independent experiments. (E, F) Western blotting to detect Bcl-2 protein with anti-Bcl-2 antibody (1∶1000). Results are expressed as mean ± SD of 3 independent experiments. ^*^P<0.05, ^**^P<0.005 compared with untreated cells.

In contrast, treatment with a specific p38 MAPK inhibitor, SB203850, markedly rescued the decrease of bioluminescence (Figure 5A and 5B) or iodide uptake (Figure 5C) by VP-16 and 5-FU compared with the treated control cells. To confirm the miRNA-16 expression level in the presence of SB203850, real time PCR was performed and the results showed that inhibitory of p38 MAPK drastically suppressed VP-16 and 5-FU-induced miRNA-16 upregulation (Figure 5D), indicating that the increased miRNA-16 expression by VP-16 and 5-FU is involved in p38 MAPK signaling pathway.

Our previous study has demonstrated that Bcl-2 protein is a direct target gene of miRNA-16 in the development of MDR in SGC7901 gastric cancer cells [11]. To test whether Bcl-2 is involved in the VP-16 and 5-FU stimulation of miRNA-16 *via* p38 MAPK pathway, we determined Bcl-2 protein level by western blot analysis. As shown in Figure 5E and 5F, VP-16 and 5-FU significantly decreased the Bcl-2 protein level by about 50% compared with the untreated control cells in the absence of SB203850. Upon treatment together with SB203850, the decrease of Bcl-2 protein was drastically attenuated, suggesting the importance of p38 MAPK activation in VP-16 and 5-FU-induced downregulation of Bcl-2 protein.

### *In vivo* monitoring of enhanced miRNA-16 expression by VP-16 and 5-FU

**Figure 6:**
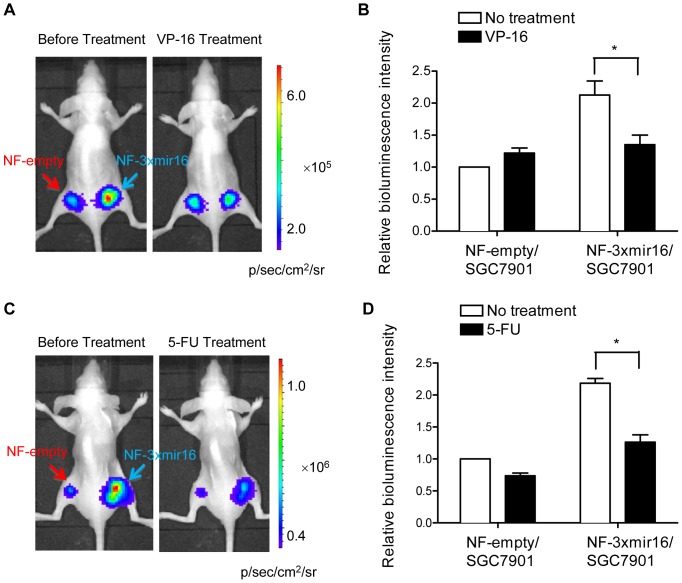
Bioluminescence imaging of enhanced miRNA-16 expression by VP-16 and 5-FU in nude mice. Different numbers of NF-empty (1×10^5^ cells) and NF-3xmir16 (1×10^7^ cells) were respectively xenografted onto the left and right hindlimb of each mouse (n = 6). Then VP-16 (A and B, 0.38 mg/kg) or 5-FU (C and D, 30 mg/kg) were intraperitoneally administrated into mice every 24 h. Before and after VP-16 or 5-FU treatment for 48 h, D-luciferin (150 mg/kg) was injected and bioluminescence imaging was acquired. Data are shown as fold changes in various groups relative to NF-empty untreated xenograft, which is set as 1. ^*^P<0.05 compared with untreated xenografts.

For noninvasive monitoring of enhanced miRNA-16 expression induced by VP-16 and 5-FU, NF-empty and NF-3xmir16 cells were grafted onto the left and right hindlimb of each mouse (n = 6), respectively. To avoid the bioluminescence signal from NF-empty interfering with that from NF-3xmir16 in the course of drug treatment, different numbers of NF-empty (1×10^5^ cells) and NF-3xmir16 (1×10^7^ cells) were grafted. As shown in Figure 6, significantly reduced bioluminescence intensity was observed in NF-3xmir16 xenografts after VP-16 or 5-FU treatment compared with the nontreatment. In particular, in mice treated with VP-16, the bioluminescence signal reduced by about 40% in NF-3xmir16 xenografts whereas increased slightly in NF-empty xenografts versus pretreated images (Figure 6A and 6B), similarly for the difference of luminescence intensity caused by VP-16 *in vitro* (Figure 4A–D). In mice treated with 5-FU, more decreased bioluminescence intensity was observed in NF-3xmir16 xenografts than in NF-empty xenografts compared with the nontreated group (Figure 6C and 6D), which was in accordance with the results induced by 5-FU *in vitro* (Figure 4A–D).

## Discussion

Drug resistance remains a major obstacle to the treatment of cancer patients for both conventional chemotherapeutic and novel biological agents. MicroRNAs have been shown to involve the management for preventing from anticancer drug resistance. In the present study, we developed a dual imaging reporter system to investigate the differential expression of miRNA-16 response to various anticancer drugs both *in vitro* and *in vivo*, and found that two clinical drugs, VP-16 and 5-FU, can upregulate miRNA-16 expression in a p38 MAPK-dependent but NF-κB-independent manner in gastric cancers.

Much effort has been exerted to study the role of miRNAs in the development of drug resistance in a variety of malignancies [7], [9], [10]. However, these trials were usually conducted *in vitro* and could not provide the *in vivo* information on the involvement of miRNAs in the drug resistance. Functional molecular imaging techniques have been emerged as a powerful tool in this regard with the advantages of favorable pharmacodynamics, target specificity and quantitative nature [19]. To monitor the kinetic expression of miRNA-16 during chemoresistance in gastric cancer cells, we introduced a dual reporter gene imaging system that enabled us to noninvasively visualize miRNA-16 by combining bioluminescence imaging and ^99m^Tc-pertechnetate gamma camera imaging. In this reporter gene system, Fluc and hNIS gene were linked to generate a fused construct. Fluc has been widely used as optical reporter gene for bioluminescence imaging of carcinogenesis, gene expression or protein-protein interactions in living subjects [20]. Although Fluc has the advantages of high sensitivity and low background activity, it is limited in terms of clinical application due to its relatively poor tissue penetration. NIS is a transmembrane protein that actively transport iodide ions into thyroid cells and could be used as a nuclear imaging reporter gene with the advantages of good tissue penetration and easy translation into clinical application [21]. While optical and nuclear imaging are complementary in many aspects, we combined Fluc and hNIS genes in a reporter gene system that can have the advantages of both modalities and would be a new avenue for basic research on monitoring miRNAs in drug resistance, as well as for early detection and diagnosis in clinic. Our results demonstrated that both the bioluminescence and radioactivity signal were correlated well *in vitro* and *in vivo*. In particular, luminescence and ^131^I uptake were decreased in NF-3xmir16 compared to NF-empty cells, whereas increased in NF-3xmi16/VCR versus NF-3xmir16 cells. *In vivo* bioluminescence and gamma camera imaging also showed decreased bioluminescence signal and ^99m^Tc-pertechnetate uptake in NF-3xmir16 than in NF-empty xenografts, whereas increased in NF-3xmi16/VCR versus NF-3xmir16 cells. These results provide evidences to suggest that both Fluc and hNIS are expressed successfully *in vitro* and *in vivo* in our reporter gene system and could reflect effectively the expression change of miRNA-16 in the chemoresistance of gastric cancer cells.

In addition, using this reporter gene system, we found that two anticancer drugs, VP-16 and 5-FU, could increase the expression level of miRNA-16 both *in vitro* and *in vivo*. And this upregulation of miRNA-16 is involved in p38 MAPK signaling pathway. The MAPK pathway is a major signal transduction cascade which ultimately triggers multiple biological cell responses following growth factor treatment or stress stimulation [22]. So far, several subfamilies of MAPKs including p38 MAPK pathway [23] and extracellular signal-regulated kinase (MEK1/2) pathway [24] have been identified. A recent study reported that inhibition of the p38 MAPK pathway sensitized human colon cancer cells to 5-FU treatment [25]. However, to date we could not find any report providing insight into the details of the role of p38 MAPK pathway in VP-16 and 5-FU-induced miRNA-16 activation. The mechanism of upregulation of miRNA-16 *via* p38 MAPK pathway may be the case that VP-16 and 5-FU increased the binding of p38 MAPK to the promoter of miRNA-16 transcript. Further studies are needed to confirm this hypothesis.

However, there are still some limitations in our reporter gene system. Though hNIS expression change was observed successfully *in vitro* experiments, *in vivo* imaging disclosed negligible activity at the site of NF-3xmir16 grafted cells. It may be due to that the weaker signal of NF-3xmir16 than NF-empty or NF-3xmir16/VCR cells and importantly the lower sensitivity of NIS reporter gene than Fluc reporter gene. So we performed only bioluminescence imaging to monitor enhanced miRNA-16 expression by VP-16 and 5-FU, but did not observe discernible difference by nuclear imaging. In addition, our reporter gene system is basically a signal-off system. In this case, it is difficult to determine whether the observed signal-off data results from the effect of miRNA expression or only from cell death *in vivo*. Therefore, further refinement of our methods is needed to improve the sensitivity and reliability of this reporter gene system.

In conclusion, we developed a dual reporter imaging system to visualize the differential expression of miRNA-16 exposure to various anticancer drugs. The reporter gene system can be used to investigate miRNA-associated molecular gennoimc events and could be used to predict the effects of chemotherapy in cancer.

## Supporting Information

Figure S1 (A) A scrambled nucleotide sequence of similar length to 3xmir16 was inserted at the 3′UTR of hNIS/Fluc fusion gene to obtain a control construct (referred to NF-scramble). Then NF-scramble, NF-empty and NF-3xmir16 vector were transfected into SGC7901 cells and investigated the Fluc gene activity by bioluminescence imaging 24 h later. (B) MTT assay to measure the growth rates of NF-empty/SGC7901, NF-3xmir16/SGC7901 and NF-3xmir16/SGC7901/VCR cell lines at 24, 48, 72 and 96 hours. (C) Quantitative RT-PCR detected Fluc gene expression in NF-empty, NF-3xmir16 and NF-3xmir16/VCR cells. Triplicate assays were performed for each RNA sample and the relative expression of Fluc was normalized to GAPDH gene. Data are shown as fold change of Fluc levels in NF-3xmir16 and NF-3xmir16/VCR relative to NF-empty cells, which are set as 1. (D, E) *In vitro* bioluminescence imaging and (F) radioiodide uptake assay after transfecting different concentrations (40, 80, 160 nM) of miRNA-16 or negative control (NC) RNA oligos (40 nM) into NF-3xmir16 cells. Imaging analysis program (Living Image software version 2.50) was used to quantify the bioluminescence intensity. Triplicate independent experiments were performed for each assay. Data are shown as fold changes in miRNA-16 transfected cells relative to NC transfected cells, which is set as 1. ^*^P<0.05, ^**^P<0.005, ^***^P<0.001compared with untreated cells. (G, H) After imaging, each of the NF-empty, NF-3xmir16 and NF-3xmir16/VCR tumors (n = 6) were collected and weighed.
(TIF)

Figure S2 **Influence of NF-κB signaling pathway on the upregulation of miRNA-16 by VP-16 and 5-FU.** NF-3xmir16 cells were pretreated with or without Bay 11-7082 (10 µmol/L) for 1 h and then incubated with VP-16 (5 µg/ml) or 5-FU (10 µmol/L) for 48 h. Then *in vitro* bioluminescence imaging (A) was performed. (B) Quantification of (A) by imaging analysis program. Results are expressed as mean ± SD of 3 independent experiments. ^*^P<0.05, ^**^P<0.005 compared with untreated cells.
(TIF)
