# Supplementary figures and images for: Noninvasive Visualization of MicroRNA-16 in the Chemoresistance of Gastric Cancer Using a Dual Reporter Gene Imaging System
Source: PLoS One. 2013 Apr 17;8(4):e61792. doi: 10.1371/journal.pone.0061792 (PMC3629136; doi:10.1371/journal.pone.0061792)

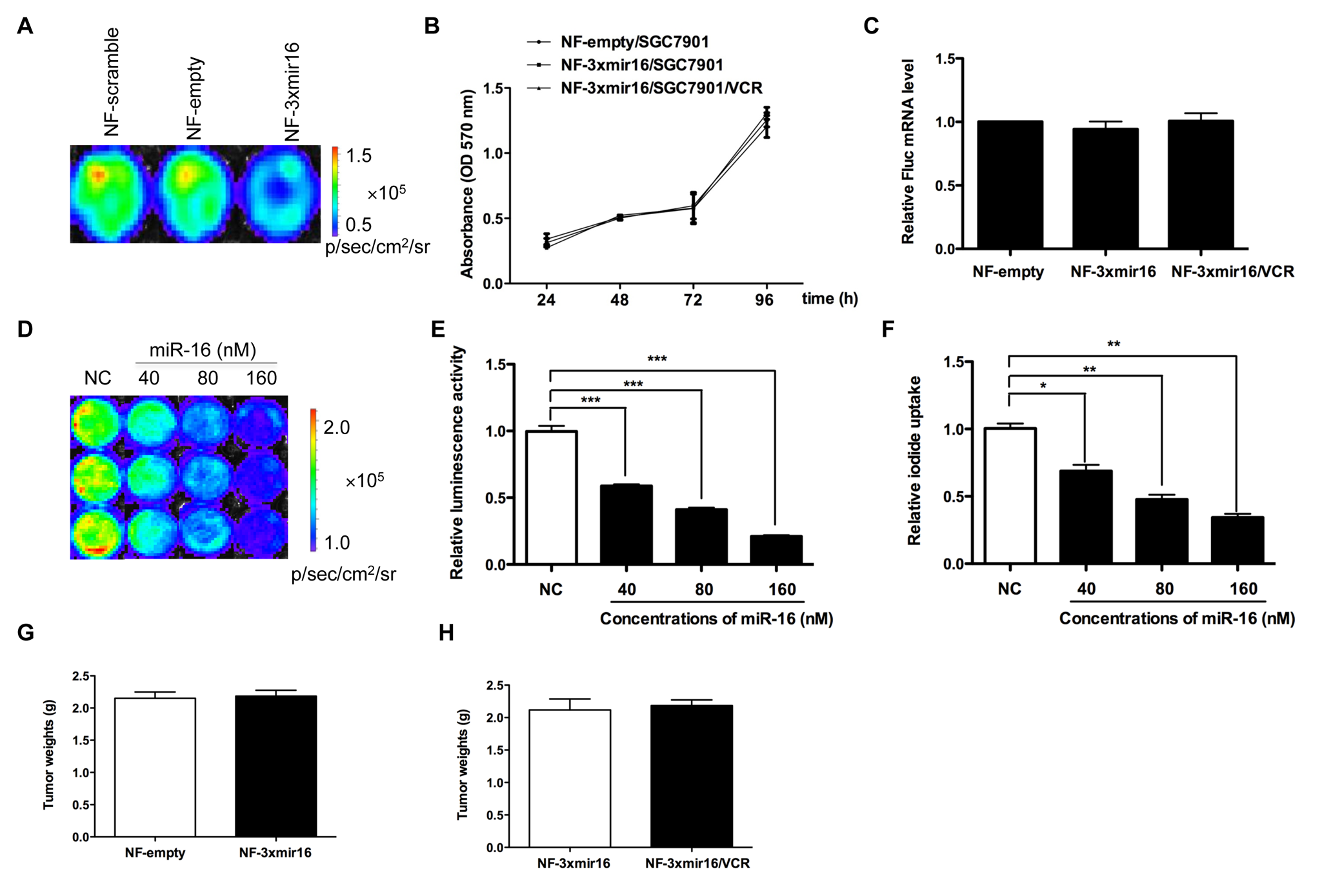

Supplement: Figure S1 — (A) A scrambled nucleotide sequence of similar length to 3xmir16 was inserted at the 3′UTR of hNIS/Fluc fusion gene to obtain a control construct (referred to NF-scramble). Then NF-scramble, NF-empty and NF-3xmir16 vector were transfected into SGC7901 cells and investigated the Fluc gene activity by bioluminescence imaging 24 h later. (B) MTT assay to measure the growth rates of NF-empty/SGC7901, NF-3xmir16/SGC7901 and NF-3xmir16/SGC7901/VCR cell lines at 24, 48, 72 and 96 hours. (C) Quantitative RT-PCR detected Fluc gene expression in NF-empty, NF-3xmir16 and NF-3xmir16/VCR cells. Triplicate assays were performed for each RNA sample and the relative expression of Fluc was normalized to GAPDH gene. Data are shown as fold change of Fluc levels in NF-3xmir16 and NF-3xmir16/VCR relative to NF-empty cells, which are set as 1. (D, E) In vitro bioluminescence imaging and (F) radioiodide uptake assay after transfecting different concentrations (40, 80, 160 nM) of miRNA-16 or negative control (NC) RNA oligos (40 nM) into NF-3xmir16 cells. Imaging analysis program (Living Image software version 2.50) was used to quantify the bioluminescence intensity. Triplicate independent experiments were performed for each assay. Data are shown as fold changes in miRNA-16 transfected cells relative to NC transfected cells, which is set as 1. *P<0.05, **P<0.005, ***P<0.001compared with untreated cells. (G, H) After imaging, each of the NF-empty, NF-3xmir16 and NF-3xmir16/VCR tumors (n = 6) were collected and weighed. (TIF) [file pone.0061792.s001.tif]

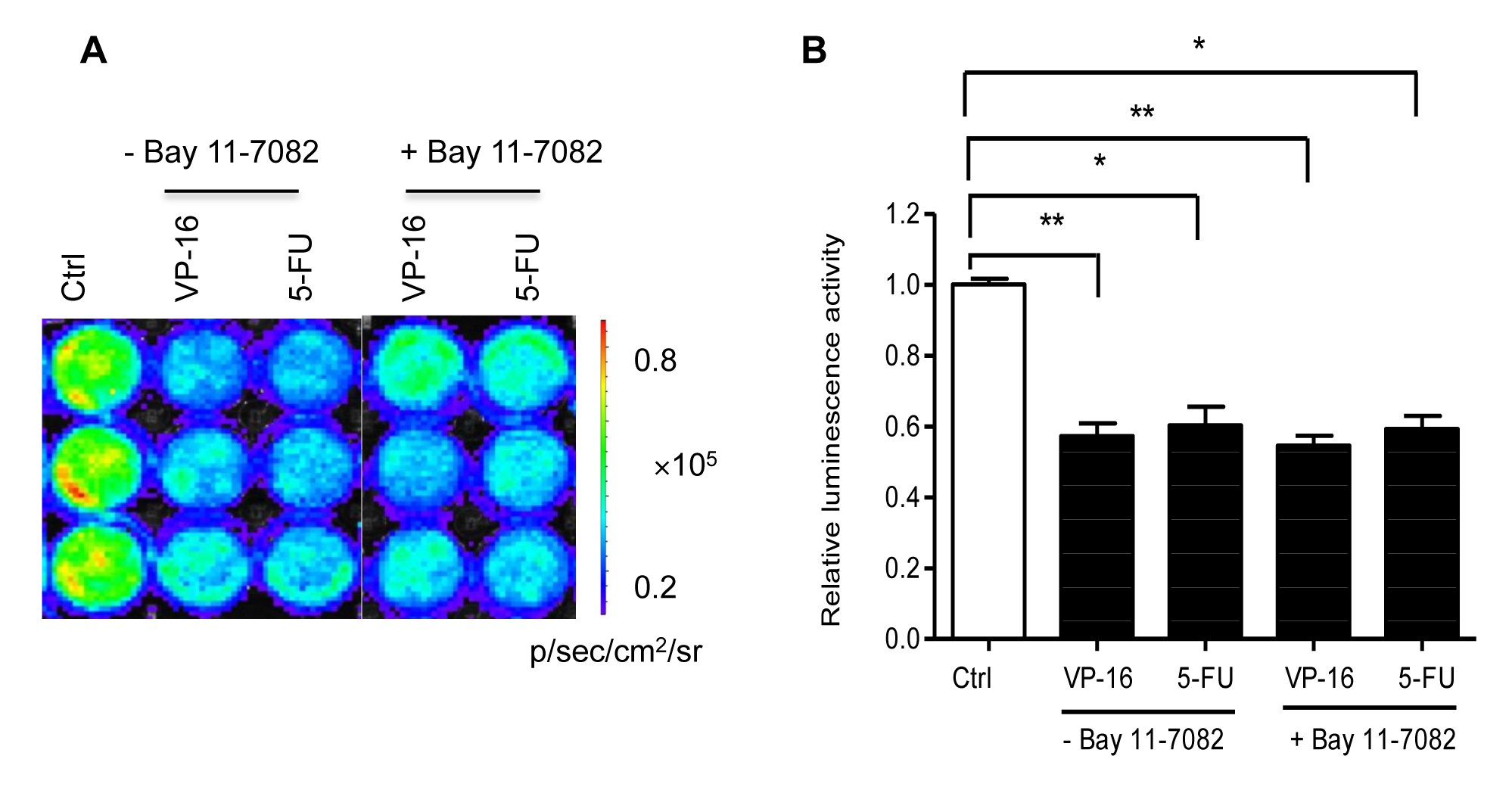

Supplement: Figure S2 — Influence of NF-κB signaling pathway on the upregulation of miRNA-16 by VP-16 and 5-FU. NF-3xmir16 cells were pretreated with or without Bay 11-7082 (10 µmol/L) for 1 h and then incubated with VP-16 (5 µg/ml) or 5-FU (10 µmol/L) for 48 h. Then in vitro bioluminescence imaging (A) was performed. (B) Quantification of (A) by imaging analysis program. Results are expressed as mean ± SD of 3 independent experiments. *P<0.05, **P<0.005 compared with untreated cells. (TIF) [file pone.0061792.s002.tif]
